# The neuroprotective activity of heat-treated human platelet lysate biomaterials manufactured from outdated pathogen-reduced (amotosalen/UVA) platelet concentrates

**DOI:** 10.1186/s12929-019-0579-9

**Published:** 2019-10-31

**Authors:** Ouada Nebie, David Devos, Valérie Vingtdeux, Lassina Barro, Jean-Christophe Devedjian, Aurélie Jonneaux, Ming-Li Chou, Régis Bordet, Luc Buée, Folke Knutson, David Blum, Thierry Burnouf

**Affiliations:** 1 Graduate Institute of Biomedical Materials and Tissue Engineering, College of Biomedical Engineering, Taipei Medical University, 250 Wu-Xing Street, Taipei, 11031 Taiwan; 2 Univ Lille, Inserm, CHU Lille, UMR-S1171. Lille Neuroscience & Cognition, Degenerative and vascular cognitive disorders, F-59000 Lille, France; 3 Univ. Lille, Inserm, CHU-Lille, UMR-S1172, Lille Neuroscience & Cognition, Alzheimer & Tauopathies, F-59000 Lille, France; 4 International Ph.D. Program in Biomedical Engineering, College of Biomedical Engineering, Taipei Medical University, Taipei, Taiwan; 5 Present address: INSERM UMRS 938, CdR Saint-Antoine, Laboratory Immune System, Neuroinflammation and Neurodegenerative Diseases, Saint-Antoine Hospital, Paris, France; 6 Clinical Immunology and Transfusion Medicine IGP, Uppsala University, Uppsala, Sweden; 7 International Ph.D. Program in Cell Therapy and Regeneration Medicine, Taipei Medical University, Taipei, Taiwan

**Keywords:** Pathogen inactivation, Intercept-platelet lysate, Ferroptosis, Neuroprotection, LUHMES cells, Primary neurons, Synaptic markers

## Abstract

**Background:**

Effective neurorestorative therapies of neurodegenerative diseases must be developed. There is increasing interest in using human platelet lysates, rich in neurotrophic factors, as novel disease-modifying strategy of neurodegeneration. To ensure virus safety, pathogen reduction treatments should be incorporated in the preparation process of the platelet concentrates used as source material. We therefore investigated whether platelet concentrates (PC) pathogen-inactivated using a licensed photo-inactivation treatment combining photosensitive psoralen (amotosalen) and UVA irradiation (Intercept) can serve as source material to prepare platelet lysates with preserved neuroprotective activity in Parkinson’s disease models.

**Methods:**

Intercept treated-PCs were centrifuged, when reaching expiry day (7 days after collection), to remove plasma and platelet additive solution. The platelet pellet was re-suspended and concentrated in phosphate buffer saline, subjected to 3 freeze-thaw cycles (− 80 °C/37 °C) then centrifuged to remove cell debris. The supernatant was recovered and further purified, or not, by heat-treatment as in our previous investigations. The content in proteins and neurotrophic factors was determined and the toxicity and neuroprotective activity of the platelet lysates towards LUHMES cells or primary cortical/hippocampal neurons were assessed using ELISA, flow cytometry, cell viability and cytotoxicity assays and proteins analysis by Western blot.

**Results:**

Platelet lysates contained the expected level of total proteins (ca. 7–14 mg/mL) and neurotrophic factors. Virally inactivated and heat-treated platelet lysates did not exert detectable toxic effects on neither Lund human mesencephalic dopaminergic LUHMES cell line nor primary neurons. When used at doses of 5 and 0.5%, they enhanced the expression of tyrosine hydroxylase and neuron-specific enolase in LUHMES cells and did not significantly impact synaptic protein expression in primary neurons, respectively. Furthermore, virally-inactivated platelet lysates tested were found to exert very strong neuroprotection effects on both LUHMES and primary neurons exposed to erastin, an inducer of ferroptosis cell death.

**Conclusion:**

Outdated Intercept pathogen-reduced platelet concentrates can be used to prepare safe and highly neuroprotective human heat-treated platelet pellet lysates. These data open reassuring perspectives in the possibility to develop an effective biotherapy using virally-inactivated platelet lysates rich in functional neurotrophins for neuroregenerative medicine, and for further bio-industrial development. However, the data should be confirmed in animal models.

**Graphical abstract:**

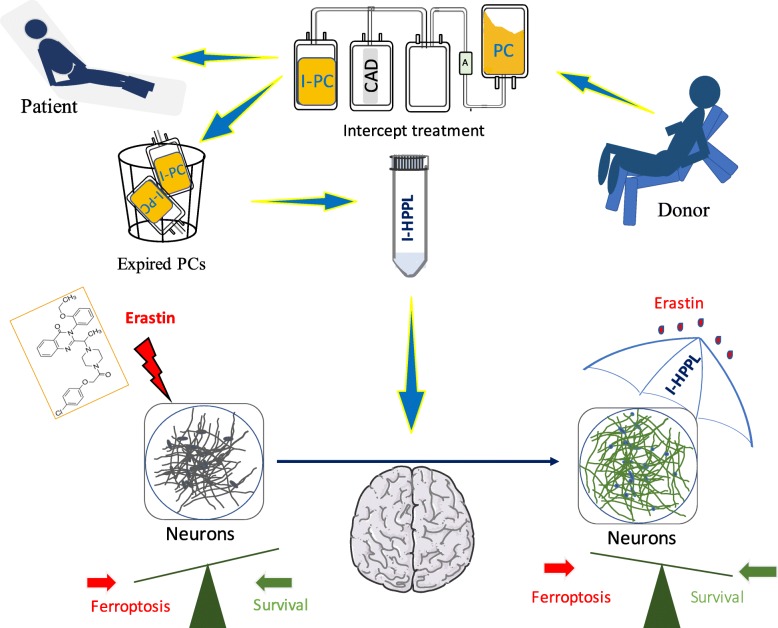

## Introduction

There is currently no licensed treatment to stimulate neurorestoration and provide neuroprotection in neurodegenerative diseases like Parkinson’s disease (PD), Alzheimer disease (AD) or amyotrophic lateral sclerosis (ALS). However, combining smart tissue engineering methods, trophic factors and advanced cell therapy may pave the way to the development of novel therapeutic strategies prone to stimulate neuronal survival, halt neuronal degeneration and thereby restore neuronal functions in patients. One promising biotherapy, currently evaluated at the pre-clinical stage, relies on the administration of human platelet lysates directly in the brain or intranasally [1–5]. Platelet lysates are rich in trophic factors including brain-derived neurotrophic factor (BDNF), platelet-derived growth factor (PDGF), vascular endothelial growth factor (VEGF), fibroblast growth factor (FGF), insulin-like growth factor I and II (IGF-I and II), transforming growth factor (TGF-β), epidermal growth factor (EGF) as well as various others cytokines, like platelet factor 4 (PF4 or CXCL4) [6]. Several studies, including ours, point-out that tailored platelet lysates exhibit neuroprotective abilities in cellular and mouse models of either PD, AD and ALS [1, 3, 7]. Pathways involved rely on PI3K/Akt, MEK and NF-κB signalings with an impact on neuroinflammation and oxidative stress [7]. Interestingly, administration of platelet lysates was also found to stimulate the proliferation of endogenous neural stem cells as well as angiogenesis, leading to reduced injury and improved functional outcomes in a stroke model [8]. Altogether, this body of evidence supports the need for further exploration of the translational value of platelet lysates to develop an optimally effective and safe biotherapy for neurodegenerative disorders [4, 5].

Platelet lysate biomaterials for regenerative medicine can be prepared from either single autologous or (unpooled/pooled) allogeneic platelet concentrates (PC). For biopharmaceutical applications, the production of platelet lysates from pooled allogeneic PC can alleviate individual donors-to-donors’ variability, due to sex, age, weight and genetic background, [9–11] and ensure optimal standardization in product specifications, including batch-to-batch consistency in neurotrophic growth factors content [12]. Although major progress has been made to ensure optimal virus safety of blood products, it remains, as shown in the past with pooled plasma products, [13, 14] that pooling increases statistically the risk of infectivity by blood-borne pathogens, most particularly viruses. Recently, a treatment using a combination of psoralen and UVA irradiation (commercialized under the name “Intercept”) has been licensed to inactivate a broad range of pathogens including viruses, bacteria, and protozoa in PCs [15, 16]. The process utilizes a photosensitive psoralen (amotosalen) that can penetrate cells and dock in-between DNA and RNA nucleic acid bases pairs, under UVA (320–400 nm) exposure [15, 17]. The chemical process leads to the establishment of an irreversible link that blocks pathogen replication [17]. Recently, it has been shown that the “Intercept” treatment, although inducing some biomolecular alterations, [18] does not substantially affect the capacity to use PC, even when reaching the expiry date for transfusion use, to prepare platelet lysates for mesenchymal stromal cell expansion [19–21] suggesting a preservation of cell growth promoting factors. However, whether psoralen/UVA treatment impacts the potential of resulting PCs for use in the context of neurodegenerative disorders remains unknown. The present in vitro study is therefore aimed at investigating whether “Intercept”-treated-PCs can be used as source material to prepare bioactive platelet lysates with preserved neuroprotective functions.

## Materials and methods

### Overall study design

**Fig. 1:**
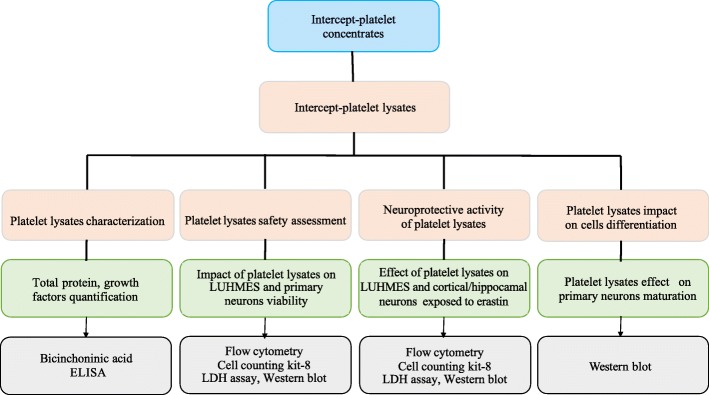
Overall study design

The experimental design is shown in Fig. 1. The main features of the platelet lysates evaluated are summarized in Table 1.

**Table 1:** Characteristics of the human platelet lysates evaluated

Full name (abbreviation)	Heat-treatment (56 °C, 30 min)	Pathogen reduction of the platelet concentrates by Intercept (amotosalen/UVA)
Intercept-platelet pellet lysate (I-PPL)	No	Yes
Intercept-heat-treated platelet pellet lysate (I-HPPL)	Yes	Yes
Heat-treated platelet pellet lysate (HPPL)	Yes	No

### Blood products preparation and characterization

#### Source of materials

Eight leukoreduced platelet concentrates (PCs) for transfusion were prepared by the blood center of the University of Uppsala, Sweden. The PCs were collected by apheresis (Trima Accel® platelet collection system, Terumo BCT, Lakewood, CO) from volunteer donors, stabilized in 35% plasma/65% platelet additive solution (SSP+) and subjected to pathogen inactivation (Cerus Corporation, Concord, CA) using “Intercept Blood System for Platelets” (150 μM psoralen (amotosalen) photosensitizer/3.9 J/cm^2^ of UVA light) [18]. The mean platelet count in such PC is 3 × 10^11^ ± 0.26 platelets/unit (Dr Knutson, personal communication). At the expiry date (7 days after collection), the PCs were centrifuged in their storage bag at 4000 x g for 30 min and the supernatant removed. The platelet pellet was frozen at − 40 °C and shipped to Taipei Medical University (TMU), Taipei, Taiwan for further processing into Intercept-platelet lysates as described below.

#### Preparation of intercept-platelet lysate (Fig. 2)

The pellets were thawed upon receipt at 35 ± 1 °C, re-suspended and concentrated in a volume of phosphate buffer saline (PBS) 1/10 that of the initial volume of PC. The suspended pellet was transferred under sterile conditions into 50-mL conical tubes, then submitted to two additional freeze-thaw (− 80 °C/37 ± 1 °C) cycles followed by centrifugation at 3000 x g, 22 ± 2 °C for 30 min. Part of the supernatant was aliquoted (I-PPL) and the rest subjected to 56 ± 1 °C for 30 min heating followed by immediate cooling for 5 min on ice to obtain I-HPPL, as we described previously [3]. A pool of eight different I-PPLs was next prepared and used as unheated material. In addition, four lots of I-HPPL (1, 2, 3, and 4) were made by pooling two I-HPPL prepared from two different PCs. Besides, a standard HPPL was prepared from a pool of 3 non-pathogen inactivated PCs collected at the Taipei Blood Center (Guandu, Taiwan) as we described previously [3] and was used as a control. All the samples were stored in aliquots at − 80 °C until use. Before all experiments, aliquots were thawed at 37 ± 1 °C and spun at 10,000 x g for 15 min at 4 ± 1 °C to remove any insoluble, and the supernatants were used for further experiments.

**Fig. 2:**
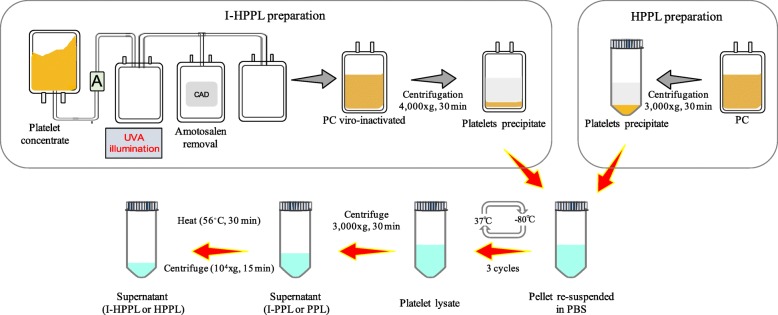
Intercept treatment and platelet lysate fractions preparation process. Abbreviations: amotosalen (A); compound absorption device (CAD); ultraviolet A (UVA); platelet concentrate (PC); phosphate buffer saline (PBS), Intercept-platelet pellet lysate (I-PPL), Intercept-heat-treated platelet pellet lysate (I-HPPL)

#### Protein content and growth factors analysis

Total protein content was measured using a bicinchoninic acid (BCA) protein assay kit (Thermo Scientific, Rockford, IL, USA). The concentrations of BDNF, EGF, PDGF-AB, and VEGF in HPPL, I-PPL, and I-HPPL were determined using a sandwich enzyme immunoassay technique (DuoSet ELISA; R&D Systems, Minneapolis, MN, USA) following the manufacturer’s protocol and as described previously [22–24].

### LUHMES cell culture and viability assays

#### LUHMES maintenance and differentiation

The Lund Human Mesencephalic (LUHMES) cell line was provided by Pr. David Devos (Department of Pharmacology and Neurology, School of Medicine, University of Lille, France). Cells were expanded and maintained in a proliferation medium: Advanced DMEM/F12 (Invitrogen, UK), 1X N-2 supplement (Invitrogen, Grand Island, NY, USA), 2 mM L-glutamine (Gibco, Rockville, MD, USA) and 40 ng/mL recombinant basic fibroblast growth factor (R&D Systems, Minneapolis, USA) in Nunclon™ cell culture flasks (Nunc, Guangzhou, China) pre-coated for 3 h with 50 μg/mL poly-L-ornithine (PLO, Sigma, St. Louis, USA) and 1 μg/mL fibronectin (Sigma). They were incubated at 37 °C in a humidified 95% air, 5% CO_2_ until confluence. To obtain differentiated cells, 2 × 10^6^ cells were seeded into 75 T flasks in proliferation medium and the differentiation was started the next day (d0), by renewing the proliferation medium with differentiation medium: Advanced Dulbecco’s modified eagle medium (DMEM/F12), 1X N-2 supplement, 2 mM L-glutamine, 1 mM cAMP (Sigma Aldrich, St Quentin Fallavier, France), 1 μg/mL tetracycline (Sigma) and 2 ng/mL recombinant glial-derived neurotrophic factor (GDNF; R&D Systems). At day 2 (d2), the cells were transferred into 24-well plate at 0.25 × 10^6^ cells per well or in 6-well plates at 1.1 × 10^6^ cells per well for an additional 3 days.

#### Safety and neuroprotective activity of intercept-platelet lysates

To investigate the potential cytotoxicity of I-PPL or I-HPPL, the LUHMES cells were cultured as described above, and cells were stimulated at day 5 of differentiation with 5% (v/v) Intercept-platelet lysates for either 24 or 48 h (Fig. 4a). For the neuroprotective effect assessment, 2.5 × 10^5^ LUHMES cells per well were seeded in 24-well plate. At day 5 of differentiation, the cells were pre-treating first with 5% platelet lysates for 24 or 48 h. When appropriate, cells were treated 1 h later by erastin (Sigma-Aldrich) at a 1.25 μM final concentration in the growth medium. In both cases, we analyzed cell viability by flow cytometry (FCM) using propidium iodide (PI, Sigma-Aldrich) staining, cell counting kit-8 (CCK-8) assay as well as proteins expression by Western blot, as described below. Lipid peroxidation was evaluated by FCM using C-11 BODIPY sensor.

#### Flow cytometry (FCM)

LUHMES cells were incubated with trypsin for 5 min, centrifuged at 500 x g for 5 min and the supernatant discarded. The pellet was next re-suspended in PBS, and the viability dye, PI (0.5 μM) was added. Lipid peroxidation was measured using 1 μM C-11 Bodipy (Life Technologies Saint-Aubin, France) according to the manufacturer’s instructions. The analysis was performed with a total of 10^4^ cells per sample using a CANTO II flow cytometer equipped with DIVA software (BD Immunocytometry Systems, San Jose, CA).

#### Cell viability assessment by CCK-8

Cell Counting Kit-8 (WST-8) Cell Proliferation Cytotoxicity Assay Kit was used according to the manufacturer’s guidelines (Sigma-Aldrich). The absorbance was measured at 450 nm, and the percentage of viable cells was expressed considering the untreated cells as 100% of control.

#### Western blot analysis

LUHMES were collected, lysed in RIPA buffer (25 mM Tris•HCl, 150 mM NaCl, 1% NP-40, 1% sodium deoxycholate, 0.1% SDS, pH 7.6, Sigma-Aldrich) buffer for 15 min on ice and sonicated (pulse: intervals 0.05 s; amplitude: 30%; and duration: 20s). Lysates were clarified by centrifugation (10,000 x g, 10 min) and the protein concentrations amounts determined using the BCA protein assay (Pierce, Rockford, IL, USA). Protein assay Samples were diluted with sodium dodecyl sulfate buffer supplemented with reducing agents (Invitrogen) and then separated on 4–12% Criterion XT Bis-Tris polyacrylamide gels (Bio-Rad, Paris, France). Proteins were transferred to nitrocellulose membranes, which were then saturated with 5% non-fat dry milk or 5% bovine serum albumin in TNT (Tris 15 mM, pH 8, NaCl 140 mM, 0.05% Tween) and incubated at 4 °C for 24 h with the primary antibodies: mouse anti-Tyrosine hydroxylase/TH (1/1000, AB152, Millipore) and anti-Neuron Specific Enolase/NSE (1/1000, NA12–47, BioMol); Anti-β-actin antibody (1/10,000, A5441, Sigma). Appropriate HRP-conjugated secondary antibodies (anti-mouse 1/50,000, A9044, Sigma; anti-rabbit 1/10,000, AP156P, Sigma) were incubated for 45 min at room temperature, and signals were visualized using chemiluminescence kits (ECL, Amersham Bioscience). Results were normalized to actin and quantifications were performed using Image J software (Scion Software).

### Neuronal cells culture and treatment

#### Primary neurons culture

A mixture of cortical and hippocampal neurons cultures was performed as described previously [25, 26]. Briefly, primary cultures were prepared from 18.5 days’ mouse embryos (C57BL/6 J) by collecting the forebrains in ice-cold media (Hanks’ balanced salt solution (HBSS)) (Invitrogen, Carlsbad, CA, USA) supplemented with 0.5% w/v D-glucose (Sigma) and 25 mM HEPES (Invitrogen). The isolation process was next done in ice-cold dissection medium in the presence of 0.01% w/v papain (Sigma), 0.1% w/v dispase (Sigma), and 0.01% w/v DNase I (Roche, Rotkreuz, Switzerland), and by incubation at 37 °C for 15 min twice. Then, the solution was spin down at 220 xg for 5 min at 4 °C. Cells were re-suspended in Neurobasal medium supplemented with 2% B-27, 1 mM NaPyr, 100 units/mL penicillin, 100 μg/mL streptomycin, and 2 mM Glutamax (Invitrogen), filtered through a 40-μm cell strainer, counted and plated on poly-L-ornithine- and laminin-coated 12-well plates at a density of 5 × 10^5^ cells/well. Fresh culture media (1:3 of starting volume) was added every 3 days until the end of the culture period. Platelet lysate treatments were applied directly in the conditioned media as described below **(**Fig. 5f**)**.

#### Cell toxicity assay and proteins expression analysis

Two types of experiments were performed on primary neuronal cultures. First, we evaluated the effect of repeated treatments with platelet lysates on the synapse maturation. In a first attempt, we investigated the potential ability of Intercept-platelet lysates to enhance or not the expression of the synaptic proteins. To do so, the analysis was performed at 14 days in vitro (DIV 14), based on the differentiation kinetic done previously [27]. For that purpose, neurons cells were seeded per well in 12-well plate and treated with 0.5% (v/v) platelet lysates (I-PPL, I-HPPL or HPPL) every 3 days starting at DIV 1 (treatments at DIV1,3,6,9,12; Fig. 5f). Synaptic markers were studied by Western blot using the following primary antibodies: anti-Munc-18 (1/1000, M2694, Sigma), SNAP25 (1/1000, Sc-376,713, Santa Cruz and SYP (H-93, sc-9116, Santa Cruz); and anti-GluA2/3/4 (1/1000, 2460S, Cell Signaling). The HRP-conjugated secondary antibodies (anti-mouse 1/50,000, A9044; anti-rabbit 1/10,000, AP156P) were purchased from Sigma-Aldrich. In a second attempt, we addressed the potential toxicity of acute platelet lysates treatments towards primary neuron’s viability with or without the presence of erastin. For these experiments, neurons were maintained for 21 days in vitro (DIV21) to ensure the development of functional neuronal networks, indicative of mature cultures. They were next treated with either 0.5% (v/v) platelet lysates (I-PPL, I-HPPL or HPPL) for 1 h followed, or not, by 1.25 μM erastin stimulation and keep for additional 2 days. The cytotoxicity was measured at DIV23 using lactate dehydrogenase (LDH) release as per the manufacturer’s instructions (CytoTox 96® Non-Radioactive Cytotoxicity Assay, Promega, Madison, WI, USA). The absorbance was acquired using a SpectraMax® i3 (Molecular Devices, Sunnyvale, CA 94089, USA) and the toxicity was calculated based on this formula: Percent cytotoxicity = 100 × (experimental LDH release (OD490)-blank (OD490))/ (LDH total (OD490)-blank (OD490)).

### Statistical analysis

Data are presented as means ± SD. Values of *p* < 0.05 were considered as indicating statistical significance by one-way analysis of variance with Fisher’s Least Significant Difference (LSD) test using GraphPad PRISM software® (GraphPad PRISM software Inc., version 8.0.0, CA, USA).

## Results

### Protein and trophic factors content of intercept-platelet pellet lysates

**Fig. 3:**
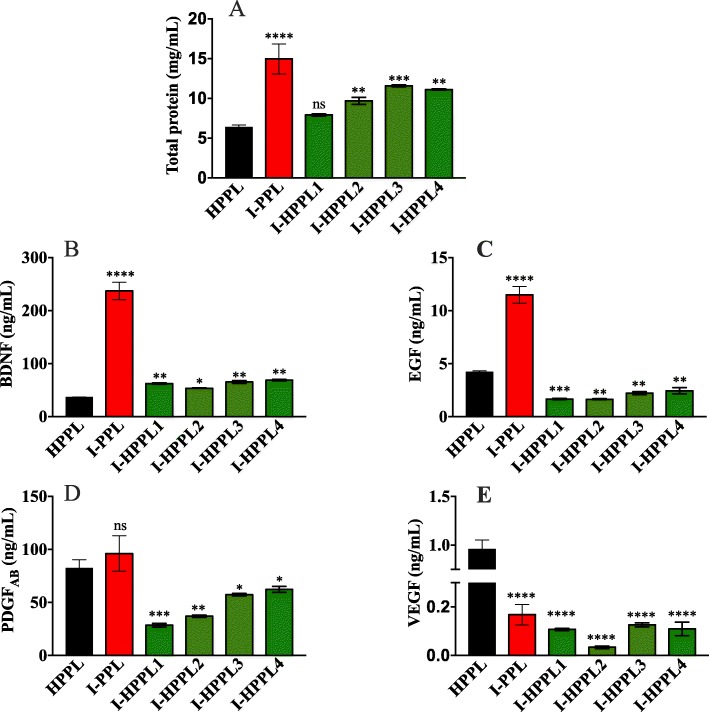
Total protein content and trophic factors in Intercept-platelet lysates. **a** Total proteins concentration (mg/ml). Concentrations in ng/ml of (**b**) brain-derived neurotropic factor (BDNF), **c** epidermal growth factor (EGF); **d** platelet-derived growth factor (PDGF)-AB, **e** vascular endothelial growth factor (VEGF). The values are expressed as the mean ± SD. I-PPL and I-HPPL were compared to the standard HPPL. ns: not statically significant. *p < 0.05; **p < 0.01 ***p < 0.001, ****p < 0.0001 vs. HPPL using One-way analysis of variance (ANOVA) followed by Fisher’s Least Significant Difference (LSD) test. Abbreviations: heat-treated platelet pellet lysate (HPPL), Intercept-platelet pellet lysate (I-PPL), heat-treated Intercept-platelet pellet lysate (I-HPPL), I-HPPL derived from pool 1 (I-HPPL1), I-HPPL derived from pool 2 (I-HPPL2); I-HPPL derived from pool 3 (I-HPPL3), I-HPPL derived from pool 4 (I-HPPL4). Each pool was prepared from 2 platelet units

Eight outdated platelet units, dedicated to transfusion and subjected to intercept treatment were used to prepare the Intercept-platelet lysates. Four pools were initially prepared, and the resulting fractions were characterized. A pool of the non-heated fractions (I-PPL) was used as a control of the heat-treated lots, whereas all the Intercept-platelet lysate fractions were also compared to standard heat-treated platelet pellet lysate (HPPL) prepared from non-virally inactivated PC. The I-PPL total protein concentration determined by BCA was 14 ± 3 mg/mL while in the heat-treated fractions (I-HPPL) the concentration ranged from 7 to 11 mg/mL. In comparison to the standard HPPL (6 mg/mL), the total proteins level in I-PPL, and most I-HPPL (2, 3, and 4, but not 1), was significantly higher (Fig. 3a). The analysis of the growth factors content by ELISA revealed a substantial amount of BDNF, EGF, PDGF-AB, and VEGF in all fractions (Fig. 3b-e). The concentrations detected in the heat-treated I-HPPL fractions were lower as compared to I-PPL and ranged from 53 to 68 ng/mL for BDNF, 1–2 ng/mL for EGF, 28–62 ng/mL for PDGF-AB, 0.03–0.1 ng/mL for VEGF. Compared to the HPPL, except for BDNF, all I-HPPL fractions showed lower EGF, PDGF and VEGF concentrations.

#### Impact of intercept-platelet lysate on cell viability and protein expression

**Fig. 4:**
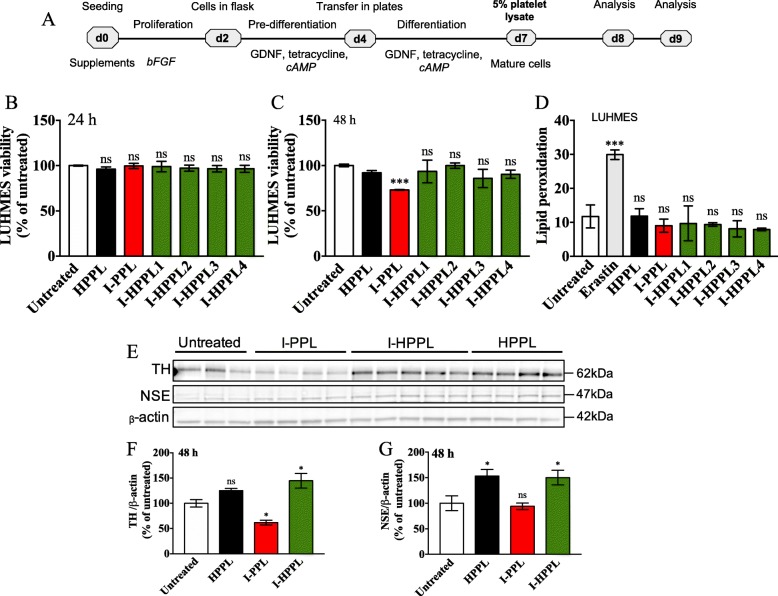
Effect of Intercept-platelet lysates on LUHMES cell viability. LUHMES cells were seeded at a density of 2.5 × 10^5^ cells/well. Following day five of differentiation, the cells were treated with 5% Intercept-platelet lysate (v/v) for 24 or 48 h. Controls were cells grown in differentiation medium only. Cells treated with Erastin only were used as a positive control for lipid peroxidation evaluation. After 24 h of incubation, the cells were collected and stained with propidium iodide for cell viability analysis or C11-Bodipy for lipid peroxidation measurement using flow cytometry. After 48 h of incubation, cell viability was assayed using cell counting kit-8 (CCK-8 test). Timeline of experiments is given in (**a**). **b** and **c** cell viability after 24 and 48 h of incubation, respectively. **d** lipid peroxidation levels. For proteins expression evaluation, the lysates of treated-cells were subjected to Western blot analysis. **e** Representative Western blots for Tyrosine hydroxylase (TH) and Neuron Specific Enolase (NSE) expression. **f** and **g** Quantifications. All data (at least *n* = 3) were expressed as the mean ± SD of untreated controls. ns: not statically significant; *p < 0.05, **p < 0.01 ***p < 0.001 vs. untreated controls using One-way analysis of variance (ANOVA) followed by Fisher’s Least Significant Difference (LSD) test. Abbreviations: day 0 (d0); heat-treated platelet pellet lysate (HPPL Intercept-platelet pellet lysate (I-PPL); heat-treated Intercept-platelet pellet lysate (I-HPPL)

To determine the potential impact of Intercept-platelet lysates on neuronal survival, we evaluated their possible toxicity on differentiated dopaminergic LUHMES cells as well as on primary neuronal cultures. Differentiated LUHMES cells were treated with the different platelet lysates (I-PPL, I-HPPL, HPPL) for 24 or 48 h (Fig. 4a). Twenty-four hours following treatment, we examined cell viability (PI) and lipid peroxidation (Bodipy) using FCM evaluations. After 48 h of treatment, cell viability was also determined using the CCK-8 test. As shown in Fig. 4b, d, and additional file 1: Figure S1 (FCM profiles) none of the fractions tested exhibited a detrimental effect on cell viability or favour lipid peroxidation in the dopaminergic LUHMES cultures after 24 h of treatment. However, following 48 h of treatment, I-PPL clearly demonstrated a significant toxic effect (26.9% ± 0.44%; *p* < 0.001 vs. control). Interestingly, Western blot analysis **(**Fig. 4e-g**)** revealed that, as compared to control untreated condition, treatment of differentiated LUHMES cells with a pool of the 4 heat-treated fractions (I-HPPL) for 2 days significantly enhanced TH and NSE protein expressions (*p* < 0.05) in a similar way than HPPL. In contrast, I-PPL toxicity was confirmed by the reduced expression of TH (p < 0.05 vs controls).

**Fig. 5:**
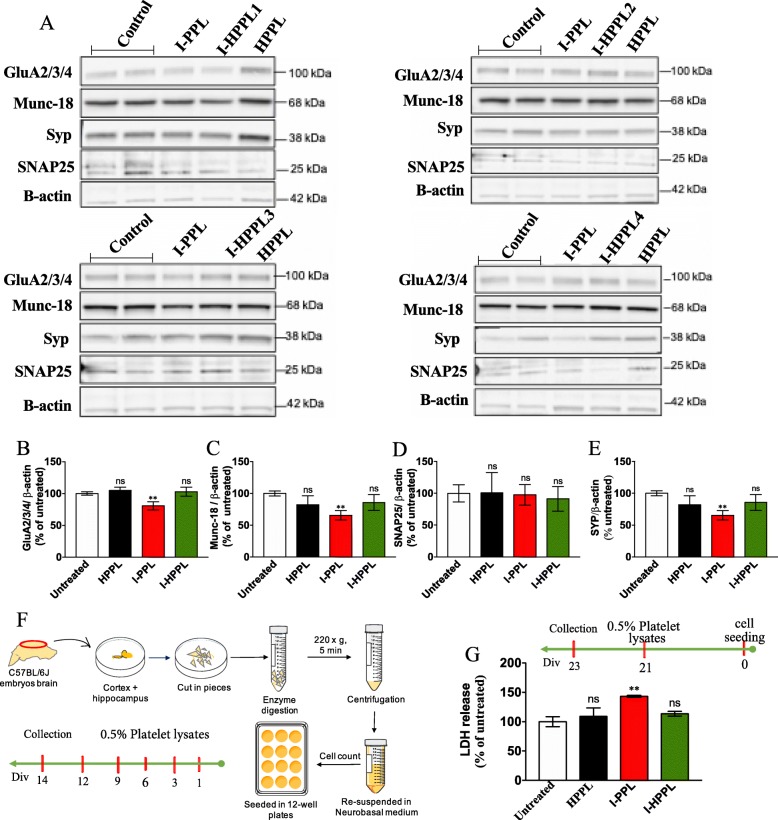
Effect of platelet lysates on primary neurons synaptic proteins expression and survival. Evaluation of platelet lysates on neuronal maturation was done as following (**a-e**). Mouse primary neurons were seeded in 12 well-plate, then treated with 0.5% (v/v) of the different platelet lysates (I-PPL, I-HPPL, HPPL) at DIV1, 3, 6, 9, 12. Whole cell lysates were prepared at DIV14 to perform Western blots to detect synaptic proteins (GluA2/3/4, Munc-18, Synatophysin or Syp and SNAP25). **a** Representative Western blot. (B-E) Densitometric analysis with synaptic protein levels were normalized to loading controls (β-actin). Data are given as averages from 4 experiments as percentage of the untreated controls. Evaluation of platelet lysate toxicity on mature neurons was performed as following. In addition, to evaluate impact of platelet lysates on mature neuron viability, at DIV21, cells were treated with 0.5% of the different platelet lysates and incubated for additional 2 days. The LDH level released, taken as a cytotoxic index, was then measured to determine the impact of the treatment on the viability of cells. **f** Schematic drawing of cells isolation method and treatment timeline. **g** Percentage of cytotoxicity of treated cells versus untreated controls. All data were expressed as the mean ± SD. ns: not statistically significant; **p < 0.01 vs. untreated controls. One-way analysis of variance (ANOVA) followed by Fisher’s Least Significant Difference (LSD) test

In addition, we next investigated the effects of Intercept-platelet lysates on primary neuron viability. To estimate the impact of lysates on various markers of the synaptic specification, we first analyzed primary neurons at DIV14, i.e. just before they exhibit mature phenotype, following a treatment with the different platelet lysates every 3 days from DIV1 (DIV1, 3, 6, 9, 12, collection at DIV14; Fig. 5f). As described in Fig. 5, repeated treatment with either I-HPPL or HPPL did not significantly alter the expression of all the pre-synaptic (SNAP25, Munc-18, Synatophysin) and post-synaptic (GluR2/3/4) markers studied as compared to controls. According to data obtained in LUHMES, I-PPL showed a detrimental effect, with reduced levels of Munc-18, Synatophysin, and GluR2/3/4 as compared to untreated controls (Fig. 5b-d). To determine the effect of acute treatment with the different lysates on mature primary neurons, the latter were treated in a mature state (i.e. DIV21) and viability evaluated 2 days later (i.e. DIV23) using LDH assay. As shown in Fig. 5g, I-HPPL and HPPL did not exert toxic effects as compared to controls (*p* > 0.05) while I-PPL significantly enhanced LDH released by neurons (*p* < 0.001).

#### Neuroprotective ability of intercept-platelet lysates

**Fig. 6:**
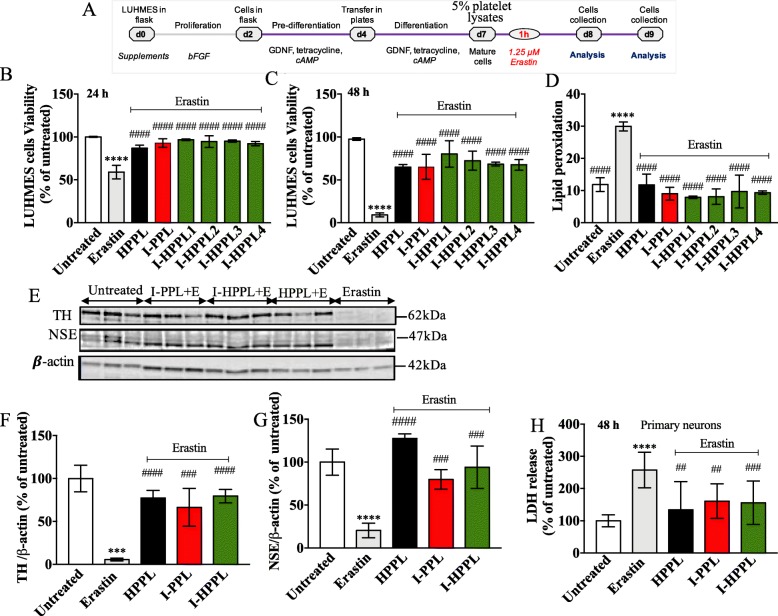
Neuroprotective ability of Intercept-platelet lysates. LUHMES cells were seeded at a density of 2.5 × 10^5^ cells/well. After day five of differentiation, cells were pre-treated with 5% Intercept-platelet lysate (v/v) for 1 h prior to the addition of Erastin and incubated for either 24 or 48 h. Controls (untreated) cells were grown in differentiation medium and treated, or not, with Erastin. After 24 h of incubation, the cells were collected and stained with propidium iodide for cell viability analysis or C11-Bodipy for lipid peroxidation measurement using flow cytometry. After 48 h of incubation, cell viability was assayed using cell counting kit-8. Timeline of experiments is given in (**a**). **b** and **c** cell viability after 24 and 48 h of incubation, respectively. **d** lipid peroxidation levels. For proteins expression evaluation, the lysates of treated-cells were subjected to Western blot analysis. **e** Representative Western blots for Tyrosine hydroxylase (TH) and Neuron Specific Enolase (NSE) expression. **f** and **g** Quantifications. All data (at least n = 3) were expressed as the mean ± SD of untreated controls. The protective effect of Intercept-platelet lysate was also evaluated using primary neurons culture. At DIV21, the mature neuronal culture was stimulated with 0.5% Intercept-platelet lysate 1 h prior to the addition of 1.25 μM Erastin. General cytotoxicity was evaluated by LDH assay (**h**). *p < 0.05, **p < 0.01 ***p < 0.001 vs. untreated controls, ^####^*p* < 0.0001 vs erastin treated cells using One-way analysis of variance (ANOVA)

Differentiated LUHMES cells were stimulated 1 h with I-PPL, I-HPPL and HPPL followed by erastin treatment (Fig. 6a). Indeed, LUHMES cells are particularly vulnerable to the programmed cell death, ferroptosis, induced by erastin and characterized by iron accumulation and huge lipid peroxidation [28]. As expected, treatment with erastin not only led to significant cell death (Fig. 6b-c) and a rise of lipid peroxidation (Fig. 6d) but also to a strong loss of TH and NSE expressions (Fig. 6e-g). Accordingly, in the presence of erastin, TH and NSE levels were found significantly higher in LUHMES cells treated with platelet fractions (Fig. 6e-g). When compared to erastin condition, all the fractions tested, even I-PPL, were able to protect significantly from erastin-induced cell death (see also Additional file 2: Figure S2 and Additional file 3: Figure S3 for FCM profiles and representative micrographs of LUHMES upon 24 h, and 48 h treatment, respectively).

Finally, the neuroprotective activity of I-HPPLs was also analyzed in mouse primary neuronal cultures. Mature neurons (DIV21) were stimulated with the different lysates for 1 h and then exposed to erastin. The release of LDH was quantified 48 h after the treatment (i.e. at DIV23). As shown in Fig. 6h, the toxicity of erastin was significantly alleviated in the presence of all types of lysate tested supporting that “Intercept” procedure does not alter the supporting and neuroprotective properties of platelet lysates.

## Discussion

Human platelet lysates made from PCs is playing increasingly important roles in the fields of cell therapy, tissue engineering, and regenerative medicine thanks to its human origin and excellent capacity to promote cell growth and tissue repair [29]. Our previous studies have provided strong evidence of the neuroprotective activity of our HPPL platelet lysate in both in vitro and in vivo models of PD [3, 7]. The HPPL was delivered in vivo by the intranasal route to by-pass the blood-brain barrier [3], as also done by another group [1, 2]. We, and others, have experimental evidence of robust neuroprotection when administering the HPPL by intracranial or intra-cerebroventricular routes. These modes of delivery allow to by-pass the blood-brain barrier and ensure on-site delivery of the neurotrophic factors [4, 8]. However, whether a pathogen reduction of the PC may alter the neuroprotective activity of HPPL is unknown.

The present study is the first to address the impact of a licensed pathogen inactivation treatment (Amotosalen/UVA; Intercept) of PC on the neuroprotective activity of human platelet lysates. Biochemical and functional studies carried on such PC have shown an only moderate impact of pathogen inactivation on platelet hemostatic functions [17, 30, 31], but no information was available on the functional preservation of neurotrophic factors. Here, we used eight Intercept treated-PCs as starting materials collected from healthy donors to prepare two types of tailor-made platelet pellet lysates (PPL and HPPL), that we previously found to exhibit neuroprotective effects in PD and ALS models [3, 7]. The cellular models used here are established to predict neuroprotective effects in animal models of PD [32–35].

Biochemical data indicated that the total protein level of the lysates made from the Intercept-PCs was not significantly affected by the treatment compared to the standard HPPL. The protein concentrations found in HPPL (prepared from 3 PCs) were 6 mg/mL, 8 mg/mL for I-HPPL, and 14 mg/mL for I-PPL. These concentrations are similar, albeit somewhat superior for I-HPPL and I-PPL, to those found in the PPL and HPPL platelet lysates prepared from standard PCs [3]. Their content in trophic factors, including BDNF, PDGF-AB, VEGF, and EGF was also assessed. The intercept-treated platelet lysates contained more BDNF and less PDGF-AB, VEGF, and EGF than standard HPPL made from fresh PC. These differences could be attributed to (a) inevitable variations in platelet growth factors among blood donors [36, 37] (b) potential impact of Intercept on platelet activation and release of some growth factors in the (discarded) plasma compartment before expiry date, or (c) to the expiry date of 7 days allowed for the pathogen-reduced PCs (which are less prone to bacterial contaminations) instead of 5 days for untreated PCs. This will deserve deepest investigations in the future. These four growth factors were selected mostly as representative biomarkers of the composition of the platelet lysates used in our study, and because they are known to support neuronal survival [38–40]. Platelet lysates contain other neurotrophic factors, such as transforming growth factor-ß, basic fibroblast growth factor, hepatocyte growth factor [3], nerve growth factor [41], and stromal cell-derived factor 1-a [5]. These factors may contribute in a synergistic way to the functional activity of the neuroprotective platelet biotherapy [4]. Besides, the platelet lysate contains high amounts (ca. 500 μg/mL) of platelet factor 4 (CXCL4) [3] that has recently been suggested to mediate neurogenesis in the hippocampal dentate gyrus [42].

To verify the safety of Intercept-platelet lysate, in vitro studies were performed using LUHMES cell line and primary neurons cultures. On the one hand, LUHMES cells, characterized as dopaminergic neuron-like cells upon differentiation, are commonly used and is the best cellular model to-date for PD [32–35]. When differentiated, these cells express several neuronal markers including TH, dopamine transporter (DAT), the vesicular monoamine transporter (VMAT-2), and exhibit significant α-synuclein levels [34]. On the other hand, the primary neuronal cultures constitute an exciting tool for the screening of neurotoxic or neuroprotective agents as they mimic better the physio-pathological situation encountered in the brain. We found that I-HPPL, in spite of being subjected to a photo-inactivation treatment using psoralen, was toxic neither to LUHMES cells nor to primary neurons. This may be explained by the fact that in the Intercept procedure, an absorption step is performed to remove the residual psoralen. In addition, our process to make the dedicated platelet lysates for brain administration includes an isolation of the platelets, further removing any residual psoralen with the discarded plasma/PAS supernatant.

The functional cellular assays showed that I-HPPL significantly enhanced the expression of two neuronal markers (TH and NSE) in LUHMES and primary neuronal cells, compared to untreated cells. This can support that the Intercept pathogen inactivation treatment, as well as the heat-treatment at 56 °C for 30 min, preserve the functional activity of the platelet growth factors. This hypothesis is supported by previous studies in which the differentiation of LUHMES in cultures requires the supplementation by exogenous functional growth factors, such as GDNF, NGF, BDNF, IGF-1, that trigger the expression of TH [34].

Our results also revealed a possible toxic effect of I-PPL after 2 days of incubation with LUHMES cells, which is not surprising. In the preparation procedure, the starting PCs were centrifuged in bags, by contrast to tubes as was done in previous experiments at laboratory scale, therefore some residual plasma/PAS remained in the bag. Thus, the I-PPL was “contaminated” by residual plasma proteins, such as fibrinogen, known to negatively affect the viability of neuronal cells [43, 44]. Thus, the toxicity of I-PPL could likely be attributed to the presence of these proteins. Moreover, Chou et al. (2017) have shown that the heat-treatment was able to remove the fibrinogen from PPL preparations and improved its neuroprotective activity, as actually observed here. The preserved viability and the expression of synaptic proteins by primary cortical/hippocampal neurons treated repetitively from DIV1 to DIV14 support the lack of toxicity of the heat-treated platelet lysates. Moreover, 7 days of treatment with the heat-treated platelet lysates (HPPL and I-HPPL) of SH-SY5Y neuroblastoma cells is non-toxic and stimulate neuronal differentiation (manuscript in preparation).

To investigate the functional properties of Intercept-platelet lysates, we used an in vitro neurotoxicity assay based on a specific form of cell death named ferroptosis. Ferroptosis is characterized by mitochondrial shrinkage and an increase of mitochondrial membrane density [45]. It has been described as one of the mechanisms involved in the pathogenesis of PD [46]. In this study, Intercept-platelet lysates were tested using a validated and commonly used LUHMES cells model [47] and a ferroptosis inducer, erastin. Erastin mediates cell death by increasing the iron deposition and lipid peroxidation [28, 45, 48, 49]. Differentiated LUHMES cells, pre-treated with 5% I-HPPL followed by erastin intoxication, showed significant reduction in cell death accompanied with low level of lipid peroxidation, similar to the standard HPPL described previously [3, 7]. I-HPPL protective activity was next assessed in mature primary (mixed cortical/hippocampal) neurons, and as expected from LUHMES experiments, I-HPPL attenuated the erastin toxicity by decreasing the release of LDH. These data, therefore, provided objective evidence that the amotosalen/UVA process and the use of expired PCs had no impact on the anti-ferroptosis capability of the platelet lysates.

In cell models of PD (LUHMES) and ALS (NSC-34), we had found the involvement of the Akt and MEK signalling pathways when cells were exposed to our standard platelet lysate [7]. Platelet-derived molecules such as the neurotrophins (BDNF, PDGF, EGF etc.), platelet extracellular vesicles, miRNAs are all potential bioactive compounds involved in many physiological events [50]. Their presence in Intercept-platelet lysate, as found here, is most likely contributing to the neuroprotective function.

The possibility to use expired Intercept-PC as source material to prepare neuroprotective platelet lysates is important as more blood establishment worldwide are implementing this pathogen inactivation process on PC [18] changing the supply pattern. Recent studies have already established that expired Intercept-treated PCs can be used as source material to prepare platelet lysate for MSC expansion, which is essential to ensure a supply of raw materials not affecting the availability for transfusion [51, 52]. The capacity to use pathogen-reduced PC raw material to make platelet lysate for clinical use is also essential in a context where pooling of 40 to 50 PCs appears preferable to provide product consistency and standardization. However, while pooling limits variability seen among blood donors [9–11, 53] it increases virus safety concerns and make dedicated virus/pathogen inactivation steps, such as Intercept, or combination of treatments needed to optimize virus safety [54]. Other virus inactivation treatments of PC to be considered for platelet lysate for regenerative medicine may include riboflavin/UV (Mirasol) [55] or short-wave UVC (Theraflex) [56]. It would be therefore also interesting to study the impact of these pathogen reduction treatments on the neuroprotective activity of HPPL. We have previously found that the heat-treatment done to prepare HPPL contributes to hepatitis C virus inactivation [3] providing an additional virus safety margin to a pooled HPPL.

## Conclusion

In conclusion, the data obtained with the LUHMES cell model and primary mouse neurons indicated that the Intercept treatment of the PCs does not impact the neuroprotective properties of the heat-treated HPPL. The toxicity of I-PPL could be avoided by the heat-treatment as observed before [3]. The platelet lysates conserved their richness in proteins and neurotrophins and could be used at different dosages to stimulate cells proliferation and maturation. Moreover, compared to the standard HPPL, the I-HPPL also exerted strong neuroprotective activity suggesting that allogeneic virally-inactivated PCs could be used as the source material to prepare a heat-treated platelet lysate with good safety profile and preserved neuroprotective activity in vitro. Further studies aiming at investigating the neuroprotection provided by I-HPPL in in vivo models will be relevant.

## Supplementary information

**Additional file 1:** **Figure S1.** Representative histograms of cell viability analysis by flow cytometry (propidium iodide staining) after 24 h. The cells were treated with 5% HPPL, 5% I-PPL, 5% I-HPPL.

**Additional file 2:** **Figure S2.** Representative histograms of cell viability analysis by flow cytometry (propidium iodide staining) after 24 h. The cells were treated with 5% HPPL + erastin, 5% I-PPL + erastin, 5% I-HPPL +erastin.

**Additional file 3:** **Figure S3.** Representative images of differentiated LUHMES 48 h after treatment with 5% I-HPPL. Example images showing cells treated with HPPL or I-HPPL + Erastin. Images taken at 10x magnification, scale bar = 100 μm.

## Data Availability

All materials are available from the corresponding authors.

## Abbreviations

AD: Alzheimer disease
ALS: Amyotrophic lateral sclerosis
BCA: Bicinchoninic acid
BDNF: Brain-derived neurotrophic factor
CAD: Compound adsorption device
cAMP: cyclic adenosine monophosphate
CXCL4: C-X-C chemokine ligand 4
DAT: Dopamine transporter
DIV: Days in vitro
DNA: Deoxyribonucleic acid
EGF: Epidermal growth factor
ELISA: Enzyme-linked immunosorbent assay
FCM: Flow cytometry
FGF: Fibroblast growth factor
GDNF: Glial-derived neurotrophic factor
GluA2/3/4: α-amino-3-hydroxy-5-methyl-4-isoxazolepropionic acid receptor subunits 2, 3, 4
HPPL: Heat-treated platelet pellet lysate
IGF-I and II: Insulin-like growth factor I and II
I-HPPL: Heat-treated intercept platelet pellet lysate
I-PC: Intercept”-treated platelet concentrates
I-PPL: Intercept platelet pellet lysate
LDH: Lactate dehydrogenase
Munc-18: Mammalian uncoordinated-18
NSE: Neuron Specific Enolase
PCs: Platelet concentrates
PD: Parkinson’s disease
PDGF: Platelet-derived growth factor
PF4: Like platelet factor 4
RIPA: Radioimmunoprecipitation assay buffer
RNA: Ribonucleic acid
SNAP25: Synaptosomal nerve-associated protein 25
SYP: Synaptophysin
TGF: Transforming growth factor
TH: Tyrosine hydroxylase
TNT: TRIS NaCl Tween 20
UVA: Ultraviolet light A
VEGF: Vascular endothelial growth factor
VMAT-2: Vesicular monoamine transporter
