# Supplementary figures and images for: The neuroprotective activity of heat-treated human platelet lysate biomaterials manufactured from outdated pathogen-reduced (amotosalen/UVA) platelet concentrates
Source: J Biomed Sci. 2019 Oct 31;26:89. doi: 10.1186/s12929-019-0579-9 (PMC6822406; doi:10.1186/s12929-019-0579-9)

Figure S1

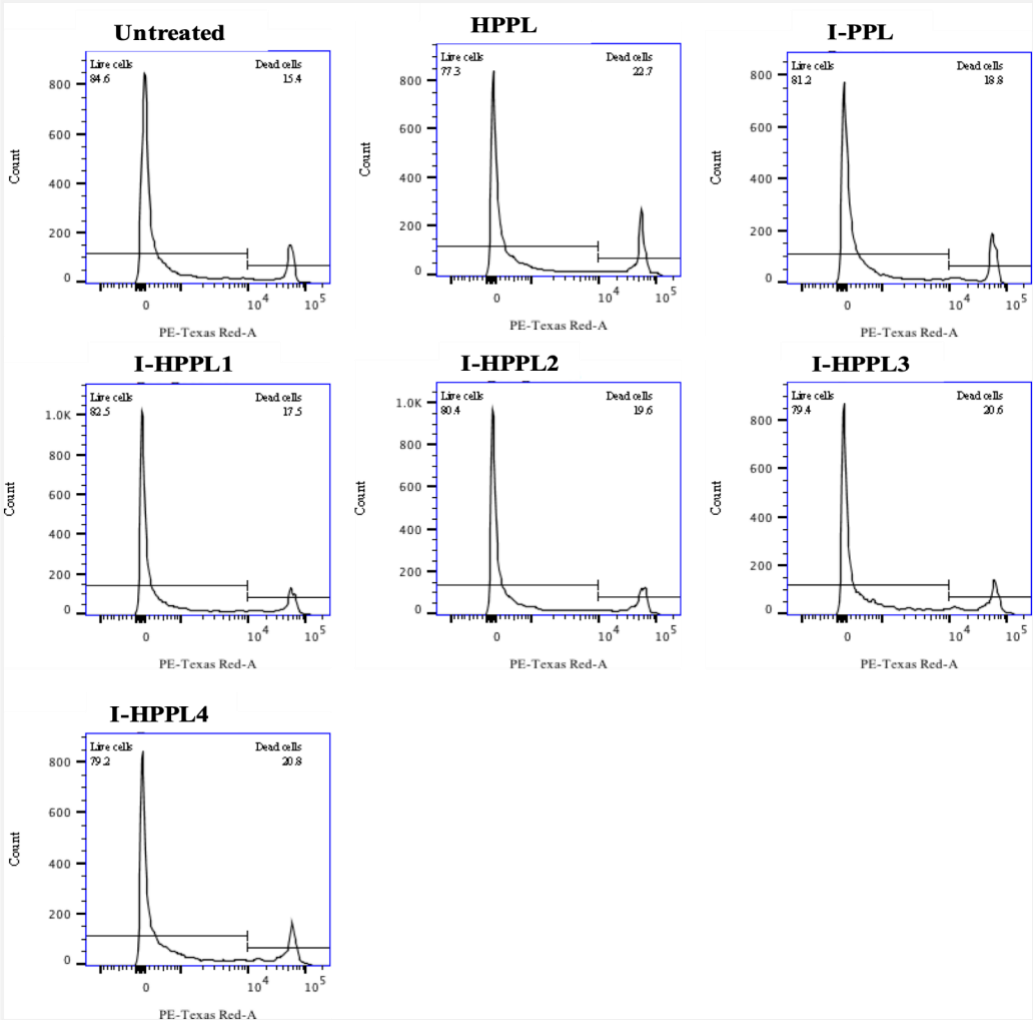

Supplement: Supplementary file 1 — Additional file 1: Figure S1. Representative histograms of cell viability analysis by flow cytometry (propidium iodide staining) after 24 h. The cells were treated with 5% HPPL, 5% I-PPL, 5% I-HPPL. [file 12929_2019_579_MOESM1_ESM.pdf]

Figure S2

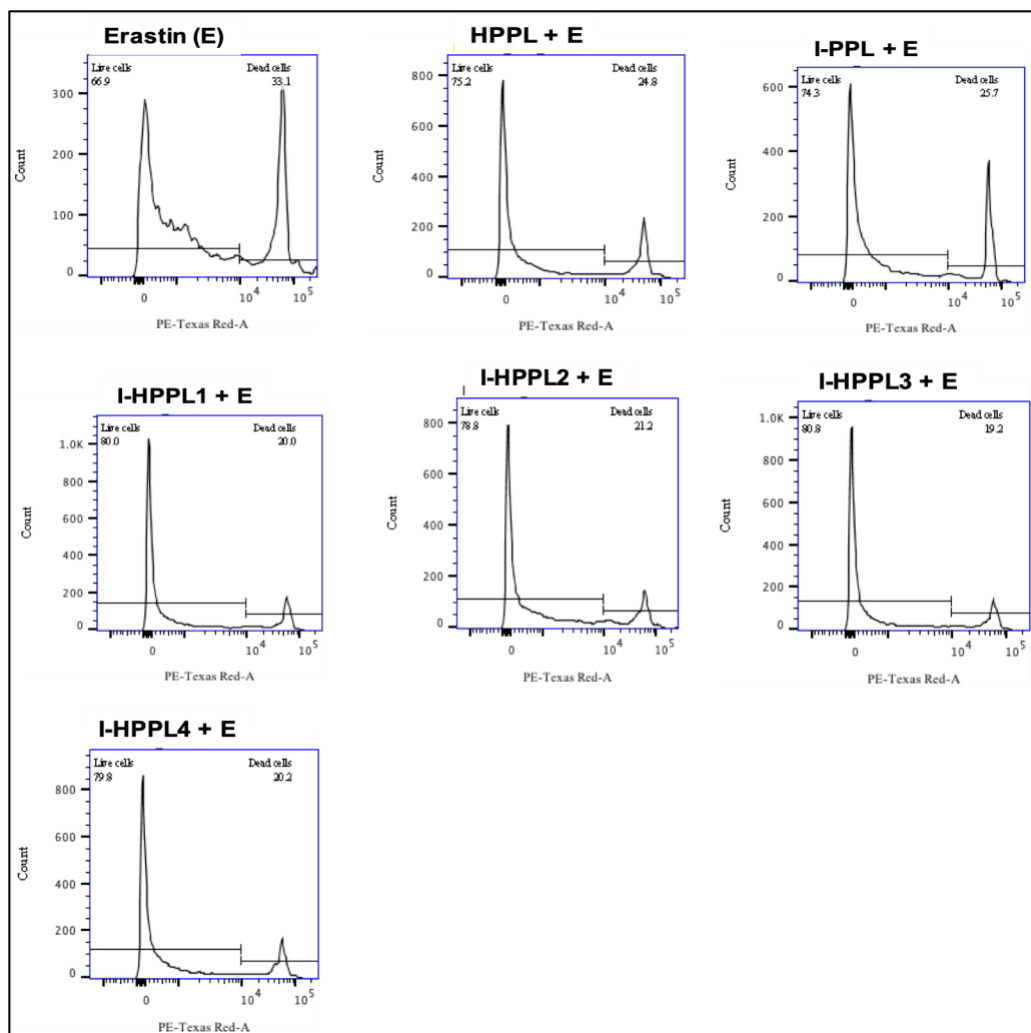

Supplement: Supplementary file 2 — Additional file 2: Figure S2. Representative histograms of cell viability analysis by flow cytometry (propidium iodide staining) after 24 h. The cells were treated with 5% HPPL + erastin, 5% I-PPL + erastin, 5% I-HPPL +erastin. [file 12929_2019_579_MOESM2_ESM.pdf]

**Figure S3**

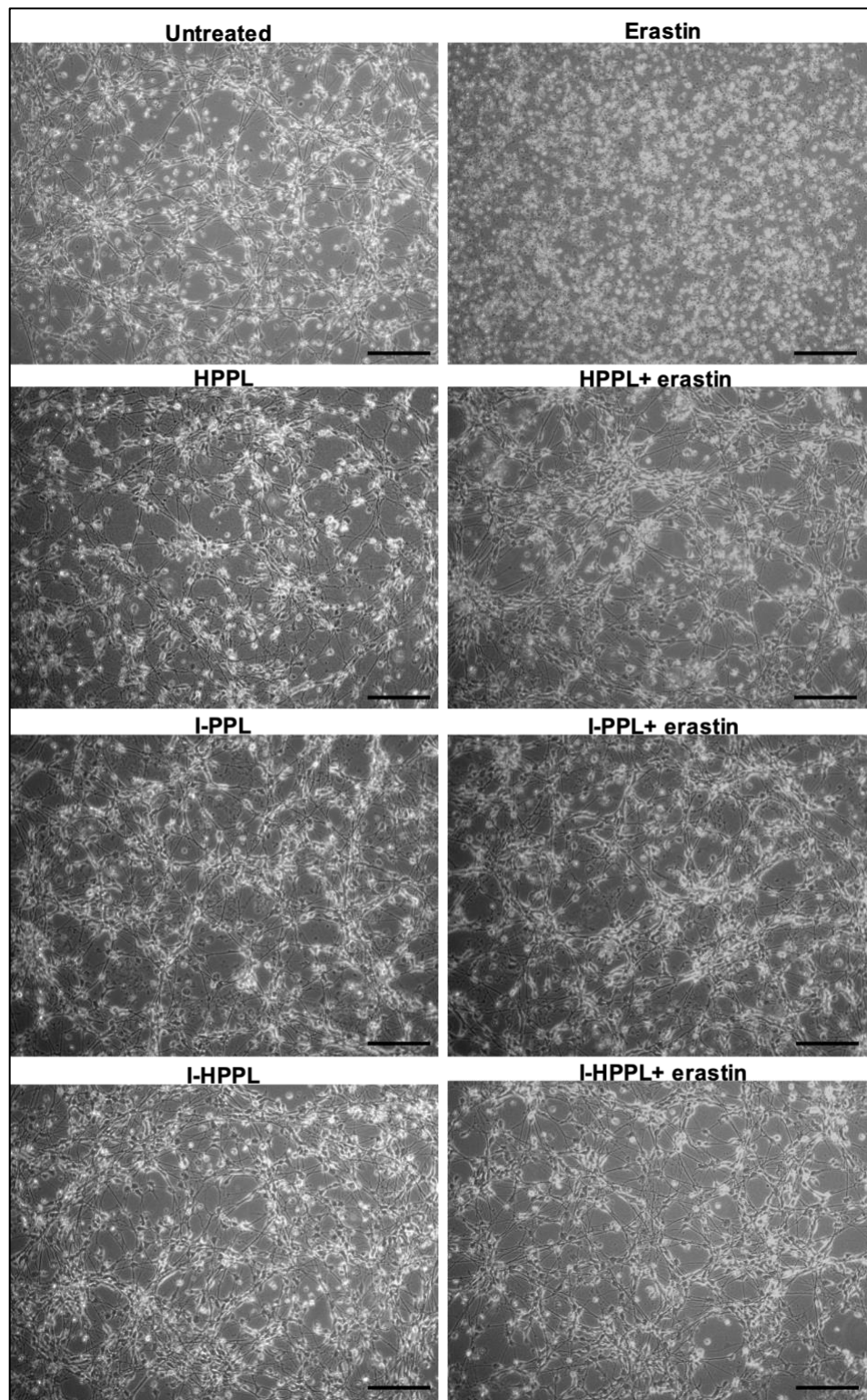

Supplement: Supplementary file 3 — Additional file 3: Figure S3. Representative images of differentiated LUHMES 48 h after treatment with 5% I-HPPL. Example images showing cells treated with HPPL or I-HPPL + Erastin. Images taken at 10x magnification, scale bar = 100 μm. [file 12929_2019_579_MOESM3_ESM.pdf]
